# Quantifying the environmental and food biodiversity impacts of ultra-processed foods: evidence from the European Prospective Investigation into Cancer and Nutrition (EPIC) study

**DOI:** 10.1017/S1368980025101067

**Published:** 2025-09-11

**Authors:** Jeroen Berden, Giles T. Hanley-Cook, Bernadette Chimera, Dagfinn Aune, Maria Gabriela M. Pinho, Geneviève Nicolas, Bernard Srour, Christopher J. Millett, Emine Koc Cakmak, Emmanuelle Kesse-Guyot, Esther M. González-Gil, Eszter P. Vamos, Jessica Blanco Lopez, Julia Baudry, Justine Berlivet, Kiara Chang, Mathilde Touvier, Charlotte Le Cornet, Chloé Marques, Christina C. Dahm, Daniel B. Ibsen, Franziska Jannasch, Guri Skeie, Maria-José Sanchez, Matthias B. Schulze, Sara Grioni, Yvonne T. van der Schouw, Ana M. Jimenez Zabala, Anna Winkvist, Anne Tjønneland, Carlotta Sacerdote, Cecilie Kyrø, Elisabette Weiderpass, Marcela Guevara, Pauline Frenoy, Rosario Tumino, Salvatore Panico, Verena Katzke, Xuan Ren, Paolo Vineis, Pietro Ferrari, Carl Lachat, Inge Huybrechts

**Affiliations:** 1 Nutrition and Metabolism Branch, International Agency for Research on Cancer, Lyon, France; 2 Department of Food Technology, Safety and Health, Faculty of Bioscience Engineering, Ghent University, Ghent, Belgium; 3 Food and Nutrition Division, Food and Agriculture Organization of the United Nations, Rome, Italy; 4 Department of Research, Cancer Registry of Norway, Norwegian Institute of Public Health, Oslo, Norway; 5 Department of Nutrition, Oslo New University College, Oslo, Norway; 6 Department of Epidemiology and Biostatistics, School of Public Health, Imperial College London, London, UK; 7 Copernicus Institute of Sustainable Development, Utrecht University, Utrecht, The Netherlands; 8 Université Sorbonne Paris Nord and Université Paris Cité, INSERM, INRAE, CNAM, Centre of Research in Epidemiology and StatisticS (CRESS), Nutritional Epidemiology Research Team (EREN), F-93017 Bobigny, France; 9 Public Health Policy Evaluation Unit, School of Public Health, Imperial College London, London, UK; 10 NOVA National School of Public Health, Public Health Research Centre, Comprehensive Health Research Center, CHRC, NOVA University Lisbon, Lisbon, Portugal; 11 Medical Research Council Centre for Environment and Health, Imperial College London, London, UK; 12 Division of Cancer Epidemiology, German Cancer Research Center (DKFZ), Heidelberg, Germany; 13 Paris-Saclay University, UVSQ, Inserm, Gustave Roussy, CESP, Villejuif, France; 14 Department of Public Health, Aarhus University, Aarhus, Denmark; 15 Steno Diabetes Center Aarhus, Aarhus University Hospital, Aarhus, Denmark; 16 Department of Molecular Epidemiology, German Institute of Human Nutrition Potsdam-Rehbruecke, Nuthetal, Germany; 17 Department of Community Medicine, UiT The Arctic University of Norway, Tromsø, Norway; 18 CIBER in Epidemiology and Public Health (CIBERESP), Madrid, Spain; 19 Escuela Andaluza de Salud Pública (EASP), 18011 Granada, Spain; 20 Instituto de Investigación Biosanitaria ibs, GRANADA, 18012 Granada, Spain; 21 Institute of Nutritional Science, University of Potsdam, Nuthetal, Germany; 22 Fondazione IRCCS Istituto Nazionale dei Tumori di Milano Via Venezian, Milan, Italy; 23 Julius Center for Health Sciences and Primary Care, University Medical Center Utrecht, Utrecht University, Utrecht, The Netherlands; 24 Ministry of Health of the Basque Government, Sub-Directorate for Public Health and Addictions of Gipuzkoa, San Sebastián, Gipuzkoa, Spain; 25 Biogipuzkoa Health Research Institute, Group of Epidemiology of Chronic and Communicable Diseases, San Sebastián, Gipuzkoa, Spain; 26 Department of Diagnostics and Intervention, Oncology, Umeå University, Umeå, Sweden; 27 Department of Internal Medicine and Clinical Nutrition, University of Göteborg, Göteborg, Sweden; 28 Danish Cancer Institute, Copenhagen, Denmark; 29 Department of Public Health, University of Copenhagen, Copenhagen, Denmark; 30 Department of Health Sciences, University of Eastern Piedmont, Novara, Italy; 31 Unit of Epidemiology, Local Health Unit of Novara, Novara, Italy; 32 International Agency for Research on Cancer, Lyon, France; 33 Instituto de Salud Pública y Laboral de Navarra, 31003 Pamplona, Spain; 34 Navarra Institute for Health Research (IdiSNA), 31008 Pamplona, Spain; 35 Hyblean Association for Epidemiology Research, AIRE ONLUS Ragusa, Italy; 36 School of Medicine, Federico II University, Naples, Italy; 37 Cancer Epidemiology and Prevention Research Unit, School of Public Health, Imperial College London, London, UK

**Keywords:** Food processing, Environmental impact, Food biodiversity, Ultra-processed foods

## Abstract

**Objective:**

While associations of ultra-processed food (UPF) consumption with adverse health outcomes are accruing, its environmental and food biodiversity impacts remain underexplored. This study examines associations between UPF consumption and dietary greenhouse gas emissions (GHGe), land use and food biodiversity.

**Design:**

Prospective cohort study. Linear mixed models estimated associations between UPF intake (g/d and kcal/d) and GHGe (kg CO_2_-equivalents/day), land use (m^2^/d) and dietary species richness (DSR). Substitution analyses assessed the impact of replacing UPF with unprocessed or minimally processed foods.

**Participants:**

368 733 participants in the European Prospective Investigation into Cancer and Nutrition (EPIC) study.

**Setting:**

Europe.

**Results:**

Stronger associations were found for UPF consumption in relation with GHGe and land use compared with unprocessed or minimally processed food consumption. Substituting UPF with unprocessed or minimally processed foods was associated with lower GHGe (8·9 %; 95 % CI: –9·0, –8·9) and land use (9·3 %; –9·5; –9·2) when considering consumption by gram per day and higher GHGe (2·6 %; 95 % CI: 2·5, 2·6) and land use (1·2 %; 1·0; 1·3) when considering consumption in kilocalories per day. Substituting UPF by unprocessed or minimally processed foods led to negligible differences in DSR, both for consumption in grams (–0·1 %; –0·2; –0·1) and kilocalories (1·0 %; 1·0; 1·1).

**Conclusion:**

UPF consumption was strongly associated with GHGe and land use as compared with unprocessed or minimally processed food consumption, while associations with food biodiversity were marginal. Substituting UPF with unprocessed or minimally processed foods resulted in differing directions of associations with environmental impacts, depending on whether substitutions were weight or energy based.

The food system’s environment impact has become a pressing concern due to its contributions to greenhouse gas emissions (GHGe), land use and biodiversity loss^(1)^. Intensive agricultural practices, especially monocultures such as maize, wheat and soya, degrade ecosystems and narrow crop diversity. Ultra-processed foods (UPF), composed largely of ingredients produced from these high-yield crops and livestock, have been indicated to have a negative impact on the environment due to their contribution to limited crop diversity and increased vulnerability to environmental pressures^(2)^. In addition, many UPF are characterised by hyperpalatability, low satiety potential and heavy marketing that can encourage overconsumption, leading to excessive food production and associated environmental pressures, while also contributing to significant public health challenges^(3,4)^.

UPF have been linked to negative health outcomes such as obesity, CVD, depressive symptoms and certain cancers^(5)^. Consequently, countries like Mexico have incorporated recommendations to limit UPF consumption in dietary guidelines^(6)^. However, the environmental impacts of UPF have received less attention, and the potential implications of substituting UPF with unprocessed or minimally processed foods remain underexplored. With diets shifting towards greater UPF consumption globally, understanding their impact on the environment is critical, particularly in terms of GHGe, land use and preservation of food biodiversity^(7–9)^. This convergence suggests that UPF-driven overconsumption represents a shared pathophysiological mechanism underlying both human and environmental health. The same hyperpalatable formulations, low satiety signals and marketing strategies that promote excessive energy intake could simultaneously drive increased food demand and production, amplifying environmental pressures. This dual pathway through overconsumption represents a novel framework for understanding how food processing impacts both human and planetary health through a common mechanism.

This study examined the relationship between dietary intake across food processing levels and environmental outcomes – specifically GHGe, land use and food biodiversity – and evaluates the potential impact of substituting minimally processed foods for UPF among adults in the European Prospective Investigation into Cancer and Nutrition (EPIC) cohort.

## Methods

### The European Prospective Investigation into Cancer and Nutrition cohort

The EPIC cohort is a large multicentre cohort examining the links between metabolic, lifestyleand environmental factors of cancer and chronic diseases. Between 1991 and 2000, over 500 000 participants aged 25–70 years were recruited across twenty-three centres in ten European countries: Denmark, France, Germany, Greece, Italy, the Netherlands, Norway, Spain, Sweden and the United Kingdom. Dietary intake at enrolment was assessed using validated, country-specific questionnaires capturing habitual consumption over the past 12 months^(10)^. In order to study associations in a disease-free population, participants with missing dietary data, extreme energy intake-to-requirement ratios, lack of follow-up or prevalent diseases at baseline were excluded. Due to administrative constraints, cohorts from Greece, Norway and Sweden were excluded, resulting in 368 733 participants (see online supplementary material, Supplemental Figure 1).

### Dietary assessment

In the 1990s, participants’ usual food intake over the previous 12 months was assessed at baseline with country-specific dietary questionnaires. Depending on the study centre, quantitative dietary questionnaires, semi-quantitative FFQ or a combination of semi-quantitative FFQ and 7-day food records were used. Data on frequencies, portion sizes or intakes in grams per day were stored in a central International Agency for Research on Cancer (IARC) database^(10)^. Post-harmonisation of dietary data was conducted, following standardised procedures (e.g. disaggregating recipes into ingredients), to obtain a standardised food list for which the level of detail is comparable between countries. The EPIC food composition database comprises more than 11 000 food and beverage items reflecting the specificities of each country.

### Exposure - Nova classification

Standardised EPIC food items were categorised by processing level using the Nova classification: Nova 1 (unprocessed or minimally processed foods, e.g. fruits, vegetables), Nova 2 (processed culinary ingredients, e.g. oils, sugar), Nova 3 (processed foods, e.g. cheese, bread) and Nova 4 (UPF, e.g. soft drinks, flavoured yoghurts). Since the Nova classification system was developed after the EPIC dietary data collection, there was some uncertainty in classifying certain food items according to their level of food processing. Therefore, three classification scenarios were developed to address this uncertainty, a lower, middle and upper-bound scenario. This study used the most probable, middle-bound scenario^(11)^. Dietary contribution from each Nova class was expressed in both grams and kcal per day, as grams reflect absolute consumption, while kcal accounts for energy density, providing complementary insights into environmental impacts.

### Outcomes - Environmental impacts and food biodiversity

Environmental outcomes were assessed using the SHARP indicators database, which estimates GHGe and land use from life cycle assessment data encompassing production, packaging, transport and preparation^(12)^. Food items were matched between the EPIC and SHARP databases using EFSA’s FoodEx2 base-term codes^(13)^. Diet-related GHGe and land use were computed for each individual by summing the amounts for all foods consumed; GHGe was expressed as kg CO_2_-equivalents per day and land use as m² per day^(12,13)^. Food biodiversity was quantified using dietary species richness (DSR), defined as the count of unique biological species consumed across foods, beverages and mixed dishes^(14)^. Composite foods were decomposed into ingredients using standard recipes and foods consumed ‘never or less than once per month’ were not considered in the DSR computation^(14)^.

### Study covariates

Sociodemographic and anthropometric covariates included in the models were: age at recruitment, BMI height, sex, educational level, smoking status at baseline, physical activity using the Cambridge index and alcohol intake.

### Statistical analysis

Consumption of the Nova classes (g/d or kcal/d) was modelled as continuous variables. Multivariable mixed linear models with random intercepts for study centres and adjustment for sociodemographic and anthropometric variables were fitted to assess associations between Nova class consumption, GHGe, land use and DSR. Additive models assessed associations of the additional consumption of a Nova class. For this, weight- and energy-based all-component models were constructed, mutually adjusting for each Nova class, to account for the total weight or energy intake^(15)^. Additionally, substitution analyses were performed, using the ‘leave-one-out’ method estimated associations of replacing a specific amount of Nova 4 with Nova 1, by keeping total intake constant^(16)^. For instance, the substitution of Nova 4 by Nova 1 in GHGe can be parameterised as:

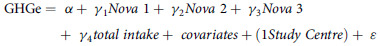




Here, *γ*_1_ represents the relative estimate for replacing a quantity of Nova 4 with an equivalent amount of Nova 1, keeping the total intake constant.

Estimates were expressed as: (1) a 1-sd increment in consumption of a Nova class, or (2) a 10 % increase from the mean absolute total dietary intake. To interpret the results as percentage differences, these estimates were divided by the mean value of the respective outcome measure.

Sensitivity analyses included baseline models only mutually adjusted for each Nova class and main models further adjusted for the Mediterranean diet score (0–18 points)^(17)^. Statistical analyses were performed in RStudio (v4.0.4.1) with two-sided testing, and *P* values < 0·05 were considered statistically significant.

## Results

### Sample characteristics

This study included 368 733 participants from the EPIC cohort, of whom 259 268 (70·3 %) were females. The mean (sd) age at recruitment was 51·3 (9·9) years, and the average BMI was 25·4 (4·3) kg/m² at baseline. On average, participants consumed 364 g (278) or 672·9 kcal (412·0) of UPF daily, representing 12·9 % (8·5) of total intake by weight and 30·5 % (15·3) by energy. Mean dietary GHGe and land use were 5·3 (1·82) kg CO_2_-equivalents per day and 6·9 (2·6) m² per day, respectively. Average DSR was 68·2 (15·2) species per year (Table 1).

**Table 1.** Baseline characteristics of 368 733 middle-aged adults enrolled in the European Prospective Investigation into Cancer and Nutrition (EPIC) study

	Mean	sd
Nova 4 (% gram per day)	12·93	8·52
Nova 3 (% gram per day)	14·28	10·56
Nova 2 (% gram per day)	1·23	1·06
Nova 1 (% gram per day)	71·56	12·50
Nova 4 (% kcal per day)	30·55	15·3
Nova 3 (% kcal per day)	25·81	12·04
Nova 2 (% kcal per day)	7·95	6·23
Nova 1 (% kcal per day)	35·70	10·62
Dietary greenhouse gas emissions (kg CO_2_ equivalents per day)	5·30	1·82
Dietary land use (m^2^ per day)	6·86	2·62
DSR (count of unique species consumed per year)	68·22	15·22
Age at recruitment (years)	51·29	9·90
BMI (kg per m^2^)	25·35	4·25
Height (cm)	165·67	8·92
	***n***	**%**
Sex		
Male	109 465	29·7 %
Female	259 268	70·3 %
Country		
Denmark	55 014	14·9 %
France	67 920	18·4 %
Germany	49 352	13·4 %
Italy	44 547	12·1 %
Spain	39 990	10·8 %
The Netherlands	36 538	9·9 %
United Kingdom	75 372	20·4 %
Education level		
None or primary school completed	102 198	27·7 %
Technical/professional school	80 266	21·8 %
Secondary school	75 288	20·4 %
Longer education (including university degree)	94 312	25·6 %
Unknown	16 669	4·5 %
Smoking status		
Never	184 435	50·0 %
Current	99 923	27·1 %
Former	78 175	21·2 %
Unknown	6200	1·7 %
Cambridge physical activity index		
Inactive	76 776	20·8 %
Moderately inactive	125 817	34·1 %
Moderately active	88 476	24·0 %
Active	70 923	19·2 %
Unknown	6741	1·8 %
Alcohol intake		
Non-drinker	44 761	12·1 %
> 0 to 6 gram per day	96 866	26·3 %
> 6 to 12 gram per day	96 048	26·0 %
> 12 to 24 gram per day	64 086	17·4 %
> 24 gram per day	66 972	18·2 %

DSR, dietary species richness. Nova 1, unprocessed or minimally processed foods. Nova 2, processed culinary ingredients. Nova 3, processed foods. Nova 4, ultra-processed foods.

### Associations between Nova class consumptions and greenhouse gas emissions, land use and food biodiversity

**Figure 1.**
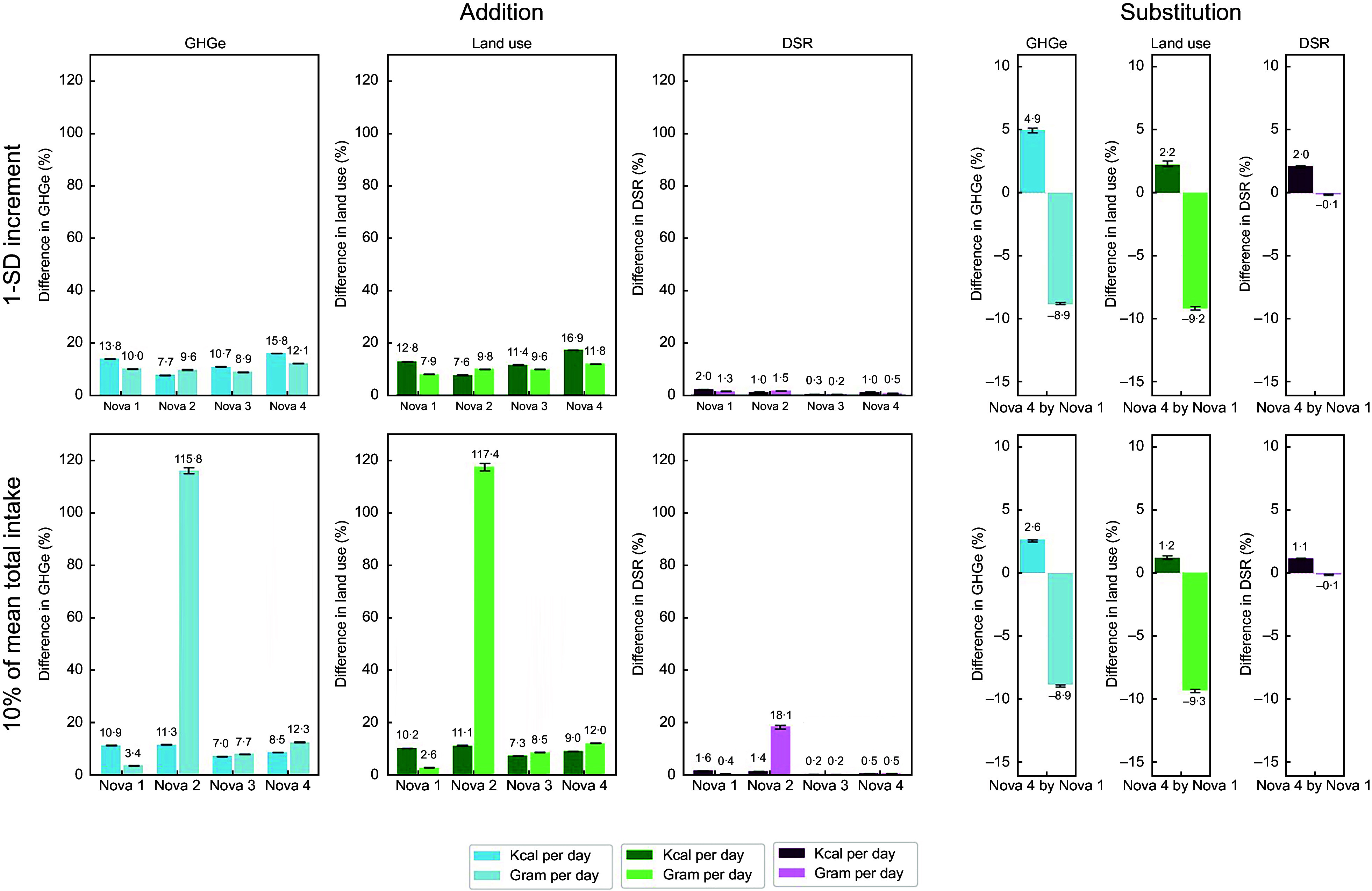
Linear association between the consumption of each Nova class and dietary greenhouse gas emissions (GHGe), land use and DSR. The left panel shows the additive estimates for 1 sd or 10 % of mean total absolute intake increase in consumption (95 % confidence intervals) across Nova classes, while the right panel presents substitution estimates for 1-sd or 10 % of mean total absolute intake substitution of Nova 4 for Nova 1 among 368 733 adults enrolled in the European Prospective Investigation into Cancer and Nutrition (EPIC) study. Nova 1: unprocessed or minimally processed foods, Nova 2: processed culinary ingredients, Nova 3: processed foods and Nova 4: ultra-processed foods. Additive models were mutually adjusted for each Nova class. Substitution models were adjusted for Nova 1, 2, 3 and total intake. Both models were also adjusted for socio-demographic and anthropometrics covariates including: age at recruitment (years), BMI (kg/m^2^), height (cm), sex (male, female), educational level (none, primary school, secondary school/technical school, higher education, unknown), smoking status at baseline (never, former, current and unknown), physical activity (Cambridge index; inactive, moderately inactive, moderately active, active and unknown) and alcohol intake (non-drinker, > 0–6, > 6–12, > 12–24 and > 24 gram per day), and centre was included as a random intercept. For consumption in kcal per day, the sds are 271·6 for Nova 1, 145·2 for Nova 2, 336·0 for Nova 3 and 394·11 for Nova 4. For consumption in gram per day, the sds are 833·3 for Nova 1, 23·5 for Nova 2, 308·1 for Nova 3 and 264·3 for Nova 4. The 10 % of the mean total absolute intake in kcal per day was 218·8 and 281·9 for total absolute intake in gram per day. Substitution models substituted 1-sd of Nova 4 with an equivalent amount of Nova 1. All *P* values < 0·001. To facilitate direct comparison, the same y-axis scale was used for both 1sd and 10 % increment estimates. This may reduce visual contrast for smaller effect sizes but improves interpretability across models.

Figure 1 illustrates the percentage difference relative to the mean of GHGe, land use and DSR associated with higher consumption of each Nova class. A 1-sd increment in consumption of each Nova class, either in gram or kcal per day, was associated with significantly higher GHGe, land use and DSR, with Nova 4 consumption being more strongly associated with GHGe and land use compared with Nova 1. To illustrate, a 1-sd increment of Nova 4 consumption in kcal per day was related to 15·8 % (95 % CI: 15·8, 16·0) higher GHGe, 16·9 % (16·9, 17·1) higher land use and 1·0 % (0·9, 1·1) higher DSR, while for Nova 1 this was 13·8 % (13·8, 14·0) for GHGe, 12·8 % (12·7, 14·0) for land use. Similar findings were reported for consumption of the different Nova classes in g/d. Strengths of associations differed within Nova 4 subgroups, with animal-based products showing the strongest positive associations with GHGe and land use, while plant-based alternatives and savoury snacks showed the weakest associations (see online supplementary material, Supplemental Table 1).

### Substitution of ultra-processed with unprocessed or minimally processed foods

10 % of the mean total intake in grams per day substitution of Nova 4 substitution with Nova 1 was associated with 8·9 % (95 % CI: –9·0, –8·9) lower GHGe and 9·3 % (–9·5, –9·2) lower land use (Figure 1). However, such a substitution was related to marginally lower DSR (–0·1 %; –0·2, –0·1). Conversely, a Nova 4 substitution with Nova 1, 10 % of the mean total intake in kcal per day, was associated with higher GHGe (2·6 %; 2·5, 2·6), land use (1·2 %; 1·0, 1·3) and DSR (1·0 %; 1·0, 1·1) (Figure 1).

Sensitivity analysis confirmed our main findings (data not shown).

## Discussion

This study found that higher UPF consumption was more strongly associated with increased dietary GHGe and land use compared with unprocessed or minimally processed foods. For DSR, associations were shown to be marginal. Energy-based substitution of UPF with unprocessed or minimally processed foods were associated with higher environmental impacts, whereas weight-based substitutions were associated with lower environmental impacts.

These discrepancies likely stem from the higher energy density of UPF. Energy-based substitutions require larger quantities of unprocessed or minimally processed foods to achieve isocaloric substitutions, potentially increasing environmental impacts^(18)^. Research suggests that individuals consuming diets high in unprocessed or minimally processed foods tend to have lower energy intake compared with those with UPF-rich diets, meaning isocaloric substitution may not fully capture these differences^(19)^. In contrast, weight-based substitutions, which emphasise food weight rather than caloric equivalence, show environmental benefits that align with UPF’ well-documented tendency to promote overconsumption through their hyper palatability, low satiety, softer textures requiring less chewing, widespread availability and lower cost per calorie, which could lead to excessive energy intake, contributing to rising obesity rates^(3,4,19)^. Such overconsumption drives higher demand for foods, amplifying environmental impacts further. Additionally, while low-impact plant-based UPF have lower environmental footprints, animal-based UPF remain highly impactful, underlining the importance of considering UPF subgroups^(20,21)^. These findings support the hypothesis that overconsumption serves as a critical link between UPF consumption and environmental harm, paralleling established mechanisms for UPF-associated health risks.

Additionally, negligible DSR differences were observed when substituting UPF with unprocessed or minimal foods, diverging from findings in Brazilian diets where UPF involved fewer species^(8)^. This discrepancy may reflect methodological differences: the Brazilian study examined species diversity within UPF products at the food system level, while our analysis assessed how individual dietary patterns relate to overall species consumption.

Our findings suggest that food biodiversity operates independently from processing level in individual diets. Substituting UPF with unprocessed foods may not increase species diversity if individuals simply consume larger quantities of the same limited set of species they already consume. Therefore, reducing UPF consumption alone may be insufficient to improve dietary biodiversity without concurrent efforts to promote species diversification. Alternatively, UPF-driven overconsumption may increase total food intake, maintaining DSR through higher consumption volumes rather than dietary diversification.

Limited observational evidence on UPF’ environmental impacts exists, with most insights coming from life cycle assessments^(22)^. In a French cohort study, it was found that UPF accounted for 19 % of energy intake in the diet and contributed to 24 % of GHGe, 23 % of land use and 26 % of energy demand. These highlight the significant environmental burden associated with diets rich in UPF, with higher contributions from post-farm stages, in particular processing regarding energy demand^(23)^. A longitudinal study showed reducing UPF consumption lowered GHGe and energy demand, but increased water use^(24)^. Our study is unique in its large, diverse European cohort, allowing a comprehensive assessment of food processing levels and substitution effects.

Several limitations must be acknowledged. The EPIC cohort may differ substantially from current European populations. UPF intake has risen dramatically – from approximately one-third of energy intake in our cohort to over half in recent studies, due to changes in food environments and consumption patterns^(25)^. Although educational attainment has increased across EU member states, this has not corresponded with expected reductions in UPF consumption, suggesting altered socioeconomic determinants of dietary choices^(26)^. The shift toward sedentary lifestyles correlates with increased convenience food reliance, while younger populations exhibit greater price sensitivity toward UPF products^(27,28)^. These transitions suggest our cohort likely underestimates the environmental impacts of contemporary European diets. Misclassification within the Nova system and reliance on SHARP database estimates, which lack country specificity and farming method variations, may introduce error. Additionally, many UPF-specific ingredients (e.g. additives) lack environmental impact assessments, and UPF typically rely on more intensively produced commodity ingredients than non-UPF, differences our analysis cannot fully capture. These errors might underestimate the true associations due to differential measurement error. Variations in dietary assessment methods and the number of items included between centres could also affect DSR, and taxonomic limitations hinder further analysis of food biodiversity. The questionnaires did not distinguish between homemade and industrianlly processed foods, which could overlook ingredient differences leading to varying environmental impacts. Lastly, this study compared individuals rather than actual substitutions, and context-specific factors such as preparation time, cost and food safety may influence dietary shifts and willingness to make substitutions^(18,29)^. For instance, while unprocessed or minimally processed foods are often more nutrient-dense, UPF offer greater accessibility and food safety^(30)^.

In conclusion, UPF consumption was more strongly associated with GHGe and land use as compared with unprocessed or minimally processed food consumption, while associations with food biodiversity were marginal. Substituting UPF with unprocessed or minimally processed foods resulted in differing directions of associations with environmental impacts, depending on whether substitutions were weight or calorie based.

## Supporting information

Berden et al. supplementary material Berden et al. supplementary material

## Data Availability

EPIC data and biospecimens are available to investigators in the context of research projects that are consistent with the legal and ethical standard practices of IARC/WHO and the EPIC Centres. The use of a random sample of anonymised data from the EPIC study can be requested by contacting epic@iarc.fr. For information on the EPIC data access policy and on how to submit an application for gaining access to EPIC data and/or biospecimens, please follow the instructions at iarc.who.int

## Supplementary material

For supplementary material accompanying this paper visit https://doi.org/10.1017/S1368980025101067
