# Supplementary material for: Quantifying the environmental and food biodiversity impacts of ultra-processed foods: evidence from the European Prospective Investigation into Cancer and Nutrition (EPIC) study
Source: Public Health Nutr. 2025 Sep 11;28(1):e164. doi: 10.1017/S1368980025101067 (PMC12722073; doi:10.1017/S1368980025101067)
Supplement: Berden et al. supplementary material [file S1368980025101067sup001.docx]

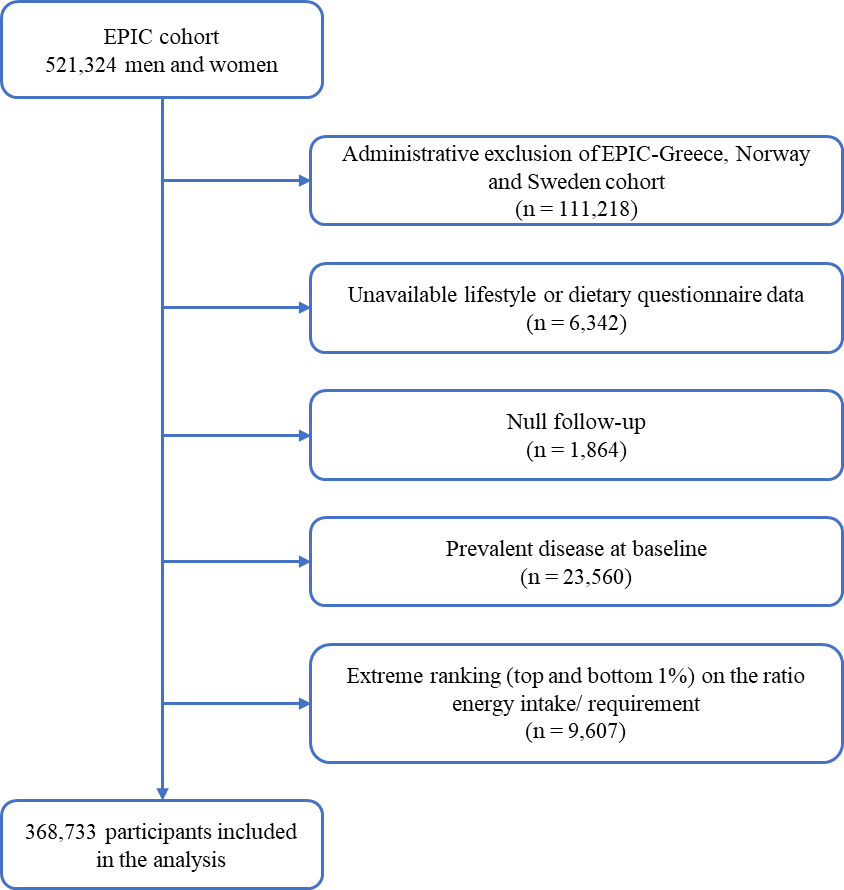
**Supplementary material**

**Supplemental figure 1. Flowchart of the participants from the European Investigation into Cancer and Nutrition (EPIC) study included in these analyses.**

**Supplemental table 1. Baseline characteristics of each Nova class and Nova 4 subgroups, and its associations with dietary greenhouse gas emissions (GHGe), land use, and dietary species richness (DSR) per 1 standard deviation (SD) increment for additive, and substitution models among 368,733 adults enrolled in the European Prospective Investigation into Cancer and Nutrition (EPIC) study.**

|  | **Mean (SD)** | | **GHGe** | | | **Land use** | | | **DSR** | | |  |
| --- | --- | --- | --- | --- | --- | --- | --- | --- | --- | --- | --- | --- |
|  | **Kcal per day** | **Gram per day** | | **Kcal per day** | **Gram per day** | | **Kcal per day** | **Gram per day** | | **Kcal per day** | **Gram per day** | |
| **Nova 1** | 764.48 (274.31) | 2,040.02 (838.96) | | 0.73 (0.73; 0.74) | 0.53 (0.52; 0.53) | | 0.88 (0.87; 0.88) | 0.55 (0.54; 0.55) | | 1.38 (1.34; 1.41) | 0.87 (0.83; 0.91) | |
| **Nova 2** | 175.09 (149.48) | 30.38 (23.56) | | 0.41 (0.40; 0.41) | 0.51 (0.51; 0.52) | | 0.52 (0.51; 0.53) | 0.67 (0.67; 0.68) | | 0.67 (0.62; 0.71) | 1.03 (1.00; 1.07) | |
| **Nova 3** | 575.93 (342.52) | 383.60 (322.76) | | 0.58 (0.57; 0.58) | 0.47 (0.46; 0.48) | | 0.78 (0.78; 0.79) | 0.66 (0.65; 0.67) | | 0.24 (0.20; 0.29) | 0.14 (0.19; 0.10) | |
| **Nova 4** | 672.88 (411.98) | 364.72 (278.19) | | 0.84 (0.84; 0.85) | 0.64 (0.64; 0.65) | | 1.16 (1.16; 1.17) | 0.81 (0.80; 0.82) | | 0.68 (0.64; 0.72) | 0.36 (0.32; 0.39) | |
| Ultra-processed breads and cereals | 100.45 (45.67) | 120.56 (50.23) | | 0.18 (0.17; 0.18) | 0.19 (0.19; 0.20) | | 0.31 (0.31; 0.32) | 0.36 (0.35; 0.36) | | 0.08 (0.03; 0.12) | 0.01 (0.04; 0.05) | |
| Sauces, spreads and condiments | 80.23 (30.12) | 85.67 (32.45) | | 0.00 (0.00; 0.00) | 0.04 (0.04; 0.05) | | 0.14 (0.13; 0.14) | 0.17 (0.16; 0.17) | | 0.00 (-0.04; 0.03) | 0.03 (0.02; 0.07) | |
| Sweets & deserts | 150.78 (60.34) | 160.12 (65.98) | | 0.51 (0.51; 0.51) | 0.44 (0.44; 0.44) | | 0.55 (0.54; 0.55) | 0.45 (0.45; 0.46) | | 0.53 (0.50; 0.57) | 0.42 (0.39; 0.45) | |
| Savory snacks | 120.56 (40.89) | 130.45 (41.20) | | 0.02 (0.02; 0.01) | 0.01 (0.01; 0.01) | | 0.01 (0.02;0.01) | 0.02 (0.02; 0.02) | | 0.15 (0.18; 0.11) | 0.12 (0.15; 0.09) | |
| Plant-based alternatives | 95.65 (22.77) | 105.32 (25.12) | | 0.02 (0.02; 0.02) | 0.00 (0.01; 0.00) | | 0.01 (0.01; 0.02) | 0.03 (0.03; 0.03) | | 0.44 (0.47; 0.40) | 0.32 (0.35; 0.29) | |
| Animal-based products | 110.12 (36.21) | 115.67 (38.40) | | 0.37 (0.36; 0.37) | 0.46 (0.46; 0.46) | | 0.53 (0.52; 0.53) | 0.63 (0.63; 0.64) | | 0.14 (0.10; 0.18) | 0.28 (0.24; 0.31) | |
| Ready to eat, heat mixed dished | 140.88 (55.44) | 150.32 (59.67) | | 0.14 (0.14; 0.15) | 0.12 (0.11; 0.12) | | 0.19 (0.18; 0.19) | 0.14 (0.14; 0.15) | | 0.40 (0.36; 0.44) | 0.21 (0.17; 0.25) | |
| Artificially, and sugar sweetened beverages | 65.34 (15.99) | 70.45 (20.89) | | 0.08 (0.08; 0.09) | 0.10 (0.10; 0.10) | | 0.06 (0.06; 0.07) | 0.08 (0.07; 0.08) | | 0.22 (0.25; 0.19) | 0.25 (0.29; 0.22) | |
| Other ultra-processed foods | 175.24 (66.89) | 180.89 (68.34) | | 0.07 (0.06; 0.07) | 0.04 (0.04; 0.04) | | 0.08 (0.08; 0.08) | 0.05 (0.05; 0.05) | | 0.05 (0.02; 0.08) | 0.04 (0.01; 0.07) | |
| **Nova 1 for Nova 4** | / | / | | 0.26 (0.25; 0.27) | -0.47 (-0.47; -0.46) | | 0.15 (0.14; 0.17) | -0.63 (-0.64; -0.62) | | 1.39 (1.32; 1.46) | -0.08 (-0.12; -0.04) | |

Nova 1: unprocessed or minimally processed foods, Nova 2: processed culinary ingredients, Nova 3: processed foods, and Nova 4: ultra-processed foods. Additive models were mutually adjusted for each Nova class. Substitution models were adjusted for Nova 1, 2, 3, and total intake. Both models were also adjusted for sociodemographic and anthropometrics covariates including: age at recruitment (years), body mass index (kg/m2), height (cm), sex (male, female), educational level (none, primary school, secondary school/technical school, higher education, unknown), smoking status at baseline (never, former, current, unknown), physical activity (Cambridge index; inactive, moderately inactive, moderately active, active, unknown), and alcohol intake (non-drinker, >0-6, >6-12, >12-24, > 24 gram per day), and centre was included as a random intercept. For consumption in kcal per day, the SDs are 271.6 for Nova 1, 145.2 for Nova 2, 336.0 for Nova 3, and 394.11 for Nova 4. For consumption in gram per day, the SDs are 833.3 for Nova 1, 23.5 for Nova 2, 308.1 for Nova 3, and 264.3 for Nova 4. Substitution models substituted 1-SD of Nova 4 with an equivalent amount of Nova 1. All P-values < 0.001, except for the numbers in grey.
